# Assessment of intracranial aneurysm rupture risk using a point cloud-based deep learning model

**DOI:** 10.3389/fphys.2024.1293380

**Published:** 2024-02-15

**Authors:** Heshan Cao, Hui Zeng, Lei Lv, Qi Wang, Hua Ouyang, Long Gui, Ping Hua, Songran Yang

**Affiliations:** ^1^ Department of Neurology, Sun Yat-sen Memorial Hospital, Sun Yat-sen University, Guangzhou, China; ^2^ Department of Cardio-Vascular Surgery, Sun Yat-sen Memorial Hospital, Sun Yat-sen University, Guangzhou, China; ^3^ Department of Biobank and Bioinformatics, Sun Yat-sen Memorial Hospital, Sun Yat-sen University, Guangzhou, China

**Keywords:** intracranial aneurysm, rupture risk, artificial intelligence, deep learning, point cloud

## Abstract

**Background and Purpose:** Precisely assessing the likelihood of an intracranial aneurysm rupturing is critical for guiding clinical decision-making. The objective of this study is to construct and validate a deep learning framework utilizing point clouds to forecast the likelihood of aneurysm rupturing.

**Methods:** The dataset included in this study consisted of a total of 623 aneurysms, with 211 of them classified as ruptured and 412 as unruptured, which were obtained from two separate projects within the AneuX morphology database. The HUG project, which included 124 ruptured aneurysms and 340 unruptured aneurysms, was used to train and internally validate the model. For external validation, another project named @neurIST was used, which included 87 ruptured and 72 unruptured aneurysms. A standardized method was employed to isolate aneurysms and a segment of their parent vessels from the original 3D vessel models. These models were then converted into a point cloud format using open3d package to facilitate training of the deep learning network. The PointNet++ architecture was utilized to process the models and generate risk scores through a softmax layer. Finally, two models, the dome and cut1 model, were established and then subjected to a comprehensive comparison of statistical indices with the LASSO regression model built by the dataset authors.

**Results:** The cut1 model outperformed the dome model in the 5-fold cross-validation, with the mean AUC values of 0.85 and 0.81, respectively. Furthermore, the cut1 model beat the morphology-based LASSO regression model with an AUC of 0.82. However, as the original dataset authors stated, we observed potential generalizability concerns when applying trained models to datasets with different selection biases. Nevertheless, our method outperformed the LASSO regression model in terms of generalizability, with an AUC of 0.71 *versus* 0.67.

**Conclusion:** The point cloud, as a 3D visualization technique for intracranial aneurysms, can effectively capture the spatial contour and morphological aspects of aneurysms. More structural features between the aneurysm and its parent vessels can be exposed by keeping a portion of the parent vessels, enhancing the model’s performance. The point cloud-based deep learning model exhibited good performance in predicting rupture risk while also facing challenges in generalizability.

## 1 Introduction

Intracranial aneurysms (IAs) are a frequently observed cerebrovascular condition, with a global prevalence estimated to be around 3.2% (Vlak et al., 2011). Although many IAs may exhibit minimal size and lack noticeable symptoms, they nonetheless carry a significant annual risk of rupture, estimated at 0.95% (Morita et al., 2012). Subarachnoid hemorrhage (SAH) is a form of hemorrhagic stroke that is associated with substantial rates of disability and mortality, and its primary cause is typically the rupture of IAs. The utilization of non-invasive imaging techniques has led to a rise in the identification of IAs. However, determining the optimal treatment for these lesions continues to be a matter of debate due to the inherent risks and complications associated with surgical clipping and endovascular coiling, especially in cases involving tiny, unruptured IAs (Wiebers et al., 2003; Brown and Broderick, 2014). The clinical decision-making process for IAs requires a delicate balance between the possible risk of rupture and the potential drawbacks of clinical intervention. Nevertheless, the traditional approaches utilized for evaluating the probability of rupture still exhibit certain limits and subjective aspects. It is therefore of tremendous clinical significance to accurately and objectively evaluate the probability of rupture in IAs in order to improve patient prognosis and overall quality of life.

Prior studies have demonstrated a connection between the rupture of IAs and several morphological factors such as aspect ratio, size ratio, and irregular shape (Ujiie et al., 2001; Kashiwazaki et al., 2013; Lindgren et al., 2016; Kleinloog et al., 2018). Additionally, clinical factors including age, hypertension, smoking, previous SAH (Greving et al., 2014; Tada et al., 2014; Can et al., 2017), as well as hemodynamic markers such as wall shear stress and oscillatory shear index (Xiang et al., 2011; Takao et al., 2012), have also been associated with IA rupture. Using statistical or machine learning (ML) techniques, many researchers have developed risk assessment models based on these factors. The authors of this dataset (Juchler et al., 2022) quantified various morphological features of aneurysms and investigated their relationship with rupture status using a LASSO regression model based on principal component analysis (PCA). However, there is currently an absence of research endeavors that focus on the development and validation of deep learning (DL) techniques.

As a critical subset of artificial intelligence, DL has exceptional proficiency in extracting nuanced features and capturing intricate relationships from extensive datasets. DL has been widely used for the purposes of detecting, predicting, and treating cerebrovascular disorders (Chen et al., 2022). There is only a tiny amount of research that has employed DL techniques for the purpose of predicting the likelihood of IA rupture. The groundbreaking work by Kim et al. (2019) involved the utilization of a DL algorithm to evaluate the likelihood of rupture in aneurysms of small dimensions (less than 7 mm). They utilized a multi-view convolutional neural network (CNN), resulting in an area under curve (AUC) of 0.755. In a study conducted by An et al. (2022), a semi-automatic ML system was built based on the CADA dataset, which consisted of 125 annotated aneurysms. The average F2-score achieved by their model, which integrates morphological, radiomic, clinical, and DL features, was 0.789. Yang et al. (2023) employed hemodynamic variables, such as wall shear stress and strain, to predict the likelihood of IA rupture. Their approach yielded an AUC of 0.883, based on a sample of 123 aneurysm cases. Ou et al. (2022) were pioneers in employing DL techniques to predict the likelihood of IA rupture based on 120 cases of stable and unstable aneurysms, rather than ruptured and unruptured aneurysms. By employing the feature fusion technique on a pre-trained model, their work achieved an AUC of 0.853. However, it is important to note that most of the studies have limited sample sizes and are based on data from single center. Consequently, there is a lack of external validation to evaluate the effectiveness of these models.

A point cloud refers a collection of points in three-dimensional (3D) space that is not arranged in any order. It serves as a representation of an object’s shape and surface features (Guo et al., 2021). Currently, several studies have been conducted to explore the utilization of point clouds across various domains. Bizjak et al. (2021) used point clouds as a predictive tool for the expansion of IAs. Chen et al. (2022) utilized hemodynamic point clouds as features and evaluated the rupture risk of IAs using machine learning algorithms. Using morphological point clouds, Li et al. (2021a), Li et al. (2021b) accurately predicted the hemodynamics before and after coronary artery bypass graft surgery, as well as Flow-Diverting Stents placement. These studies demonstrated the various applications of point clouds. However, to the best of our knowledge, no empirical research has been reported to demonstrate the efficiency of point cloud-based morphological models in predicting the likelihood of IA rupture. Point cloud can be utilized for 3D visualization in the context of aneurysm, enabling the representation of spatial contour and morphological characteristics. We hypothesize that the utilization of 3D point clouds as input for DL facilitates the neural network’s ability to comprehend a greater amount of dimensional information and examine the 3D morphological features of aneurysms from various perspectives and depths. Consequently, the primary objective of this study is to assess the feasibility and efficacy of a point cloud-based DL model for predicting the likelihood of IA rupture. This will offer a more objective and accurate reference for clinical decision-making regarding IAs.

## 2 Methods

### 2.1 Dataset description

The AneuX morphology database is an open-access and multi-centric database that has 3D geometric models of 750 IAs. These models were gathered from three distinct projects: HUG (Juchler et al., 2022), @neurIST (Villa-Uriol et al., 2011) and Aneurisk (Aneurisk-Team, 2012). The HUG initiative is a prospective and continuous effort to recruit patients, which builds upon the data collection framework established by the @neurIST project. Aneurisk, on the other hand, is an independent undertaking that relies on retrospective data and does not specify imaging timing in different stages of aneurysm development. As a result, Aneurisk was not included in this study.

The AneuX morphology database employs a standardized processing architecture (Berti et al., 2010), to extract 3D models from 3D rotational angiography (3DRA). In addition to presenting the original mesh resolution, the database also offers cleaned and re-meshed versions with target mesh cell areas of 0.01 and 0.05 mm^2^, correspondingly. In addition, the aneurysms were segmented from the entire vessels using four distinct planar and nonplanar cutting configurations, specifically referred to as dome, ninja, cut1 and cut2. We recommend consulting the original publication for a more comprehensive understanding of the database and associated processing methods (Juchler et al., 2022).

**FIGURE 1:**
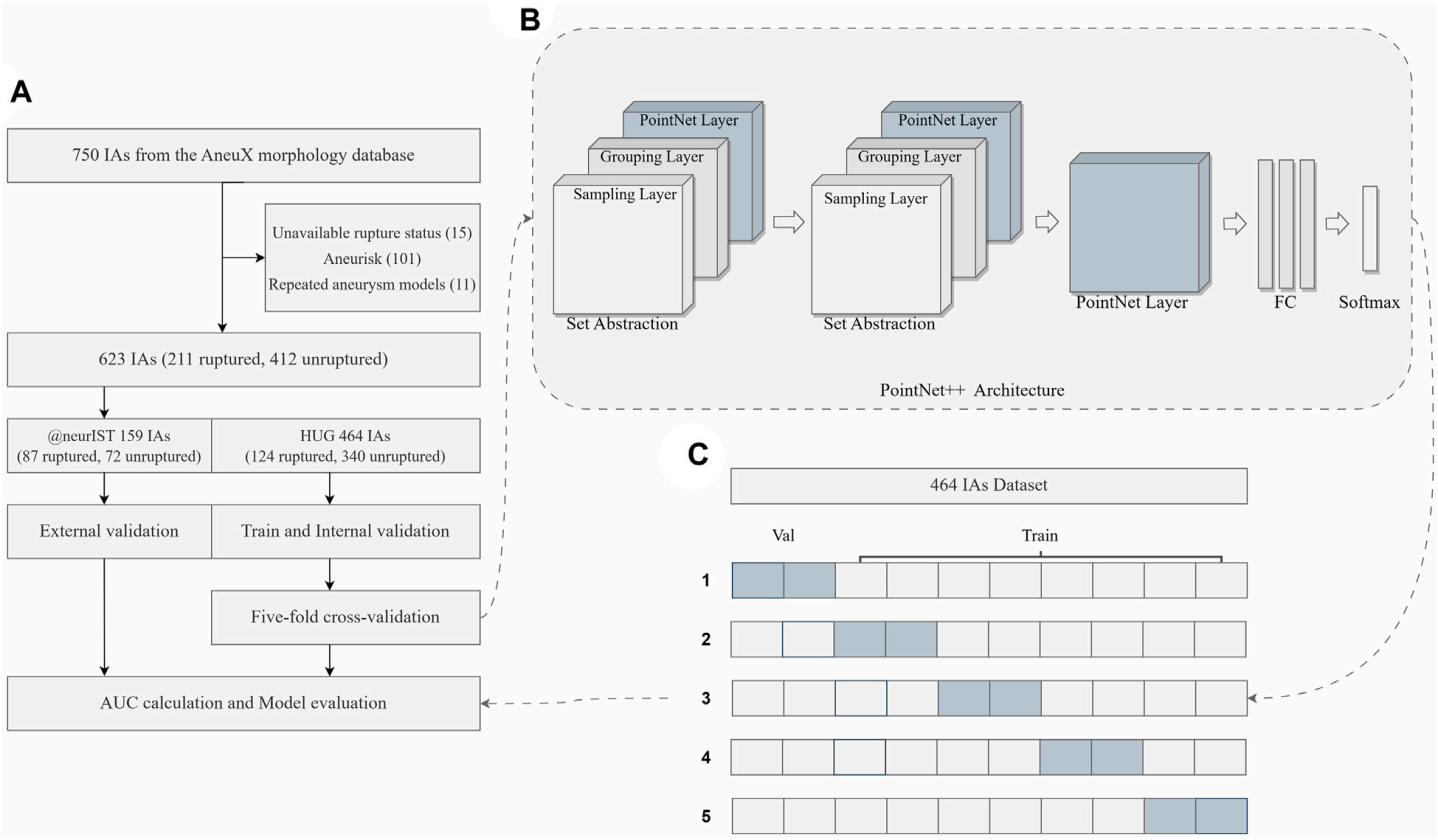
Overview of the study. **(A)** The flowchart of the dataset inclusion and exclusion, as well as model training, validation and evaluation. **(B)** The simplified framework of PointNet++ architecture. **(C)** The diagram of five-fold cross-validation. FC Layers, fully connected layers.

In this study, a total of 623 aneurysms (211 ruptured and 412 unruptured) were obtained from the HUG and @neurIST projects, as described earlier. The HUG project, consisting of 124 ruptured and 340 unruptured cases, was utilized for the purpose of model training and internal validation, while the @neurIST project, including 87 ruptured and 72 unruptured cases, was used for external validation. The inclusion and exclusion procedure is illustrated in detail in Figure 1A, and the baseline characteristics of the dataset included in this study are presented in Table 1. For further details concerning the numbering of the 15 aneurysm models with unknown rupture status and the 11 recurrent IAs, please refer to the Supplementary Material.

**TABLE 1:** Baseline characteristics of patients with ruptured and unruptured aneurysms from HUG and @neurIST projects.

	HUG		@neurIST		Total	
	R (124)	U (340)	R (87)	U (72)	R (211)	U (412)
Age	54.7 ± 14.3	55.5 ± 12.9	51.8 ± 11.7	54.0 ± 9.9	53.4 ± 13.3	55.2 ± 12.4
Female	83	268	60	55	143	323
Location						
ICA	10	124	16	32	26	156
MCA	13	104	20	22	33	126
PComA	26	29	37	15	63	44
AComA	54	41	1	0	55	41
PC	13	28	10	2	23	30
ACA	8	14	3	1	11	15

R, ruptured; U, unruptured; ICA, internal carotid artery; MCA, middle cerebral artery; PComA, posterior communication artery; AComA, anterior communication artery; PC, posterior circulation; ACA, anterior cerebral artery.

### 2.2 Data preprocessing

In this study, we assessed the effectiveness of two models in predicting the likelihood of IAs rupture. The first model exclusively considered the aneurysm dome, whereas the second model incorporated a segment of the parent arteries beside the aneurysm dome. The dome models were constructed using the segmented original models obtained from the database. A single planar incision was used to separate these models from the parent vasculature (Juchler et al., 2022). With regard to the models featuring partial preservation of the parent vessels, we followed the vessel length principle as outlined in the original article’s “cut1” method. This involved positioning the cutting surface perpendicular to the local centerline within one vessel diameter from the dome. We viewed the 3D models and noticed that some of the previously segmented cut1 models in the database did not preserve the inflow artery, which we believe contains necessary morphological information for DL feature extraction. As a result, we re-segmented the cut1 models from the original vessels, adhering to the previously mentioned cutting principle, and referred to them as cut1 models as well.

**FIGURE 2:**
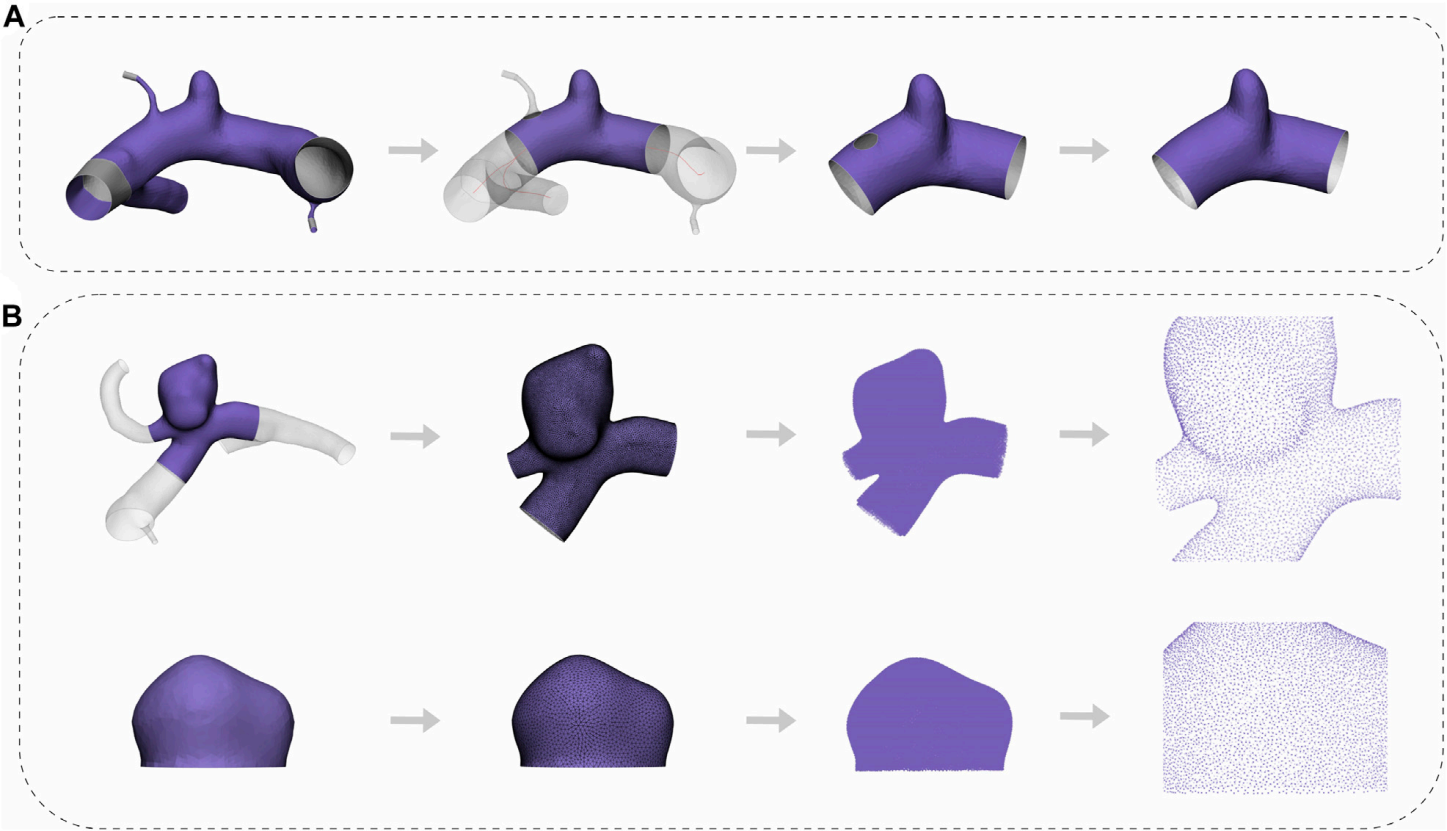
Flowchart of the pre-processing. **(A)** The whole process from cropping to repairing. **(B)** The complete process of edge smoothing, mesh reconstruction, transformation, and visualization of the aneurysm point cloud for both the cut1 model and the dome model.

The original vessel model files were imported into Mimics Medical software (version 21.0, Materialise, Leuven, Belgium), and the “Fit Centerline” function was used to autonomously generate the centerline of the vessels. Afterwards, we conducted manual measurements of the parent vessels’ diameter and performed excision of a length corresponding to the diameter of the parent vessels along a section perpendicular to the vessel centerline. In order to simplify the DL model, the small vessel branches surrounding the parent vessels were eliminated. Moreover, following the excision of a small vessel branch, a discernible notch would remain on the parent vessel. To maintain the structural integrity and ensure its continuity, we performed a restorative procedure using the “fix” function within the 3-Matic Medical software (version 13.0, Materialise, Leuven, Belgium). Figure 2A depicts the flowchart outlining the cutting process employed in the production of cut1 models.

Following the segmentation of all cut1 models, some irregular cells and uneven meshes remained, which could potentially compromise the extraction quality of point clouds. To mitigate these defects, we applied a consistent processing approach to both the dome and the cut1 models. This approach involved a moderate smoothing of sharp edges and mesh reconstruction. The “smooth” function within the 3-Matic Medical software was employed, with the smoothing factor and number of iterations configured to 0.5 and 3, respectively. To reconstruct the mesh, we used the “Uniform Remesh” tool, and the target triangle edge length was specified as 0.15. Following these procedures, the meshes were exported as stereolithography files and then converted into point cloud data in txt format using the Open3D package in Python. A flowchart of this process, applicable to both the dome and cut1 models, is shown in Figure 2B.

### 2.3 Construction of model

The PointNet architecture (Qi et al., 2017a) represents a significant breakthrough in the domain of DL for point cloud processing. Its key strength is its ability to handle unordered and irregular point sets. Nonetheless, PointNet’s performance is limited due to its inability to effectively capture local features and inter-point interactions, especially in complex point clouds that exhibit diverse local densities. PointNet++ (Qi et al., 2017b) was developed as a solution to these limitations, expanding upon the strengths of PointNet while augmenting its performance even more.

Therefore, PointNet++ was chosen as the foundational DL framework for this study. It employs a hierarchical architecture that incorporates multiple levels of set abstraction, initially abstracting small local regions before progressing to larger ones. The hierarchical structure that emerges from this process effectively captures both regional and global features. Figure 1B depicts a simplified architecture of PointNet++. In particular, each set abstraction level is comprised of three distinct layers, namely, the sampling layer, the grouping layer, and the PointNet layer. The sampling layer discerns a subset of input points to serve as the centroids of local regions. Next, the grouping layer employs a ball query strategy to identify points near the centroids, hence facilitating the construction of local region sets. To represent regional patterns as feature vectors, the PointNet layer utilizes a mini-PointNet structure. Furthermore, PointNet++ introduces a multi-scale grouping (MSG) strategy for extracting and concatenating features from various scales at the centroids of local regions. This enriches the model with multi-scale information, resulting in a more robust feature representation.

The binary cross-entropy loss function (de Boer et al., 2005) was employed for the PointNet++ model in this study. By computing the difference between the predicted class probabilities and the corresponding ground truth labels, this function normalizes the network output into a probability distribution across different classes and calculates the cross-entropy loss value. Such a value plays a crucial role in updating the network weights during training, ensuring that the predicted class probabilities align more accurately with the ground truth labels.

The training and validation of the model were carried out on a computer server equipped with an Nvidia GeForce GTX 3070 GPU. The PointNet++ code was utilized through the PyTorch framework and Python 3.8. The final hyperparameters were determined by ablation experiments conducted with a fixed random seed (Reimers and Gurevych, 2017). Specifically, a total of 8,192 points were sampled for each aneurysm using the farthest point sampling (FPS) strategy. In order to reach the requisite number of samples, FPS employed an iterative process whereby the point that is farthest from the previously selected points is successively picked as the next representative sample. The process ensures that the selected samples are evenly distributed and accurately reflect the characteristics of the whole point cloud. The training procedure utilized a batch size of 20, 200 epochs, an Adam optimizer with a weight decay of 1e-04, and an initial learning rate of 2e-05. Additionally, the Cosine Annealing Warm Restarts scheduler was utilized to reduce the learning rate through the cosine annealing method prior to each restart cycle. This approach sped up the convergence pace while minimizing the risk of overfitting.

### 2.4 Model and risk evaluation

A stratified five-fold cross-validation approach (Pedregosa et al., 2011) was employed to fully utilize the limited dataset for training and evaluating of the model, making sure that each fold contained an equivalent proportion of samples from each class. This strategy effectively prevents potential pitfalls associated with class imbalance. The receiver operating characteristic (ROC) curve was systematically plotted, and a comprehensive array of statistical indices, including accuracy, sensitivity, specificity, and AUC, was calculated for each epoch in the internal validation set. Furthermore, the ROC curve that demonstrated the highest level of reliability and robustness throughout the training set, internal validation set, and external validation set for each fold was identified as the optimal curve (Table 2). The performance of the model was assessed using the definitive evaluative metric, which was computed by taking the arithmetic mean of these optimal curves using the numpy 1.23.5 package (Harris et al., 2020).

**TABLE 2:** Performance comparison of the dome and cut1 models.

Model	Pattern	Val AUC	Accuracy	Sensitivity	Specificity	Test AUC
PointNet++ (dome)	Fold 1	0.80	0.75	0.60	0.81	0.67
	Fold 2	0.77	0.77	0.64	0.82	0.69
	Fold 3	0.84	0.82	0.64	0.88	0.70
	Fold 4	0.80	0.81	0.52	0.91	0.72
	Fold 5	0.83	0.82	0.67	0.87	0.67
	**Average**	**0.81**	**0.79**	**0.61**	**0.86**	**0.69**
PointNet++ (cut1)	Fold 1	0.81	0.83	0.80	0.84	0.71
	Fold 2	0.82	0.80	0.64	0.85	0.68
	Fold 3	0.89	0.85	0.68	0.91	0.72
	Fold 4	0.89	0.81	0.72	0.84	0.70
	Fold 5	0.84	0.82	0.75	0.84	0.73
	**Average**	**0.85**	**0.82**	**0.72**	**0.86**	**0.71**

The bold values is represent the average performance of the two models (dome and cut1) on the validation and test sets during five-fold cross-validation.

**FIGURE 3:**
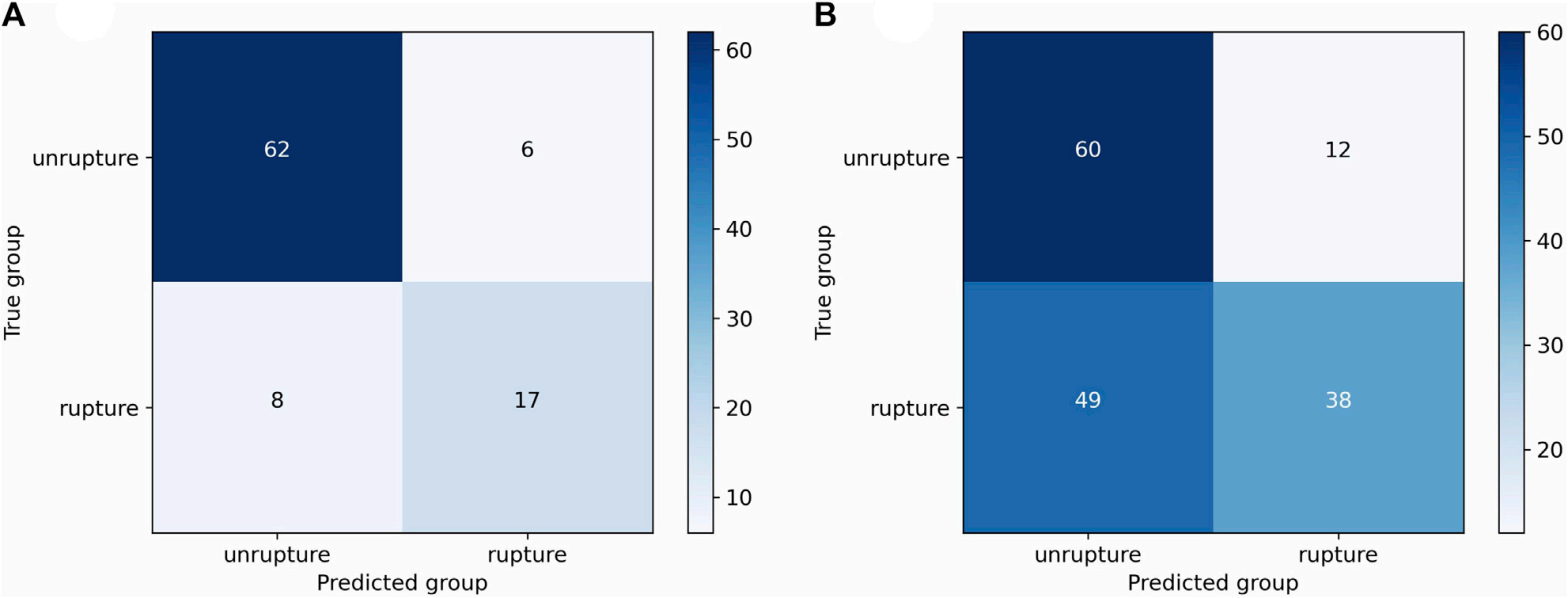
Confusion matrices of the cut1 model’s third fold on internal and external validation sets. **(A)** Confusion matrix on internal validation set. **(B)** Confusion matrix on external validation set.

**FIGURE 4:**
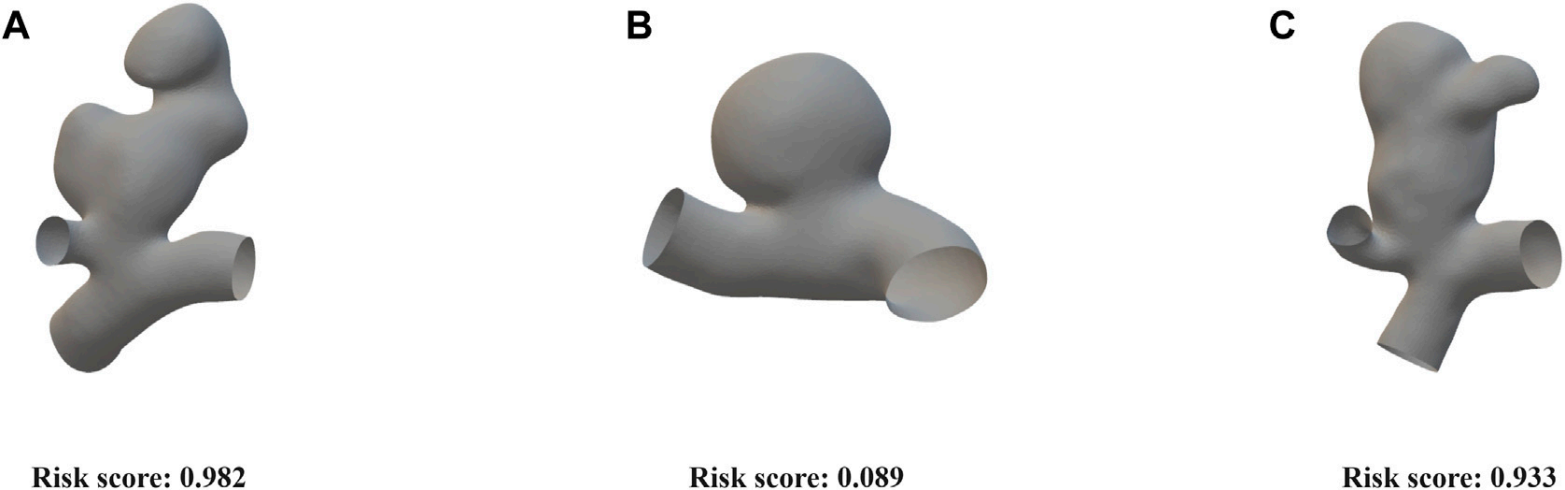
Risk scoring diagram generated by the cut1 model’s third fold on the external validation set. **(A)** An irregularly shaped ruptured aneurysm with a significant bulge, exhibiting large aspect ratio and size ratio. Our model assigned a risk score of 0.982. **(B)** An elliptical-shaped, unruptured aneurysm with a regular morphology. Our model assigned a risk score of 0.089. **(C)** An irregularly shaped, unruptured aneurysm with a significant bulge, exhibiting large aspect ratio and size ratio. Our model assigned a risk score of 0.933.

The model (cut1, fold3) that demonstrated greater overall performance was chosen for the evaluation of rupture risk. The confusion matrices of this model on both the internal validation set and external validation sets were illustrated in Figure 3. Upon processing point cloud data derived from an IA, the final output layer of the model would produce a set of rupture risk scores, transformed by a softmax layer (Goodfellow et al., 2016). These scores, which ranged from 0 to 1, served as an indicator of the likelihood of the IA rupture. A probability value trending towards 1 indicated an increased likelihood of IA rupture, whereas a value closer to 0 signified a decreased likelihood of IA rupture. Risk scoring diagram for the external validation set was illustrated in Figure 4.

## 3 Results

**FIGURE 5:**
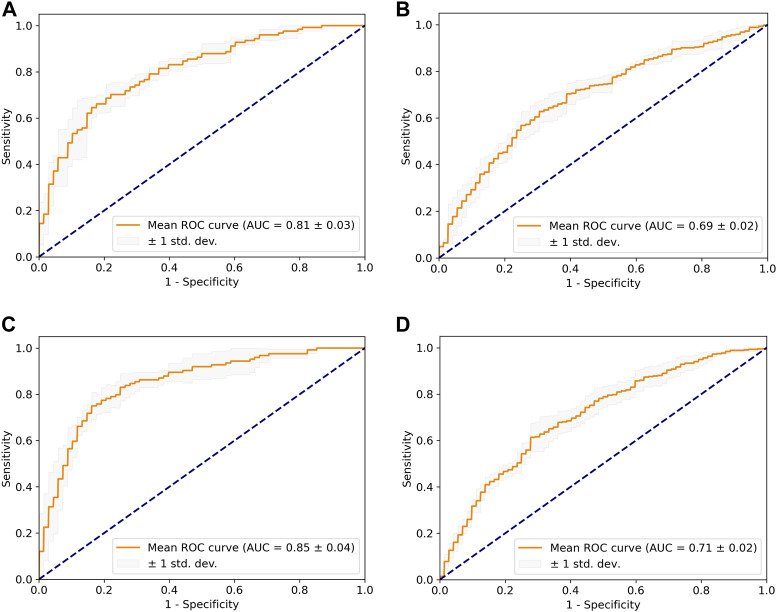
The mean ROC curve based on five-fold cross-validation. **(A)** Dome + internal validation. **(B)** Dome + external validation. **(C)** Cut1 + internal validation. **(D)** Cut1 + external validation.

Two models, namely, the dome model and the cut1 model, were constructed using the AneuX morphology database. The HUG project in the database consisted of 211 ruptured aneurysms and 412 unruptured aneurysms, which were partitioned in a 4:1 ratio for training and internal validation using stratified five-fold cross-validation. Table 2 listed the evaluation metrics used to quantify the models’ performance, and Figure 5 depicted the mean ROC curves. The dome model exhibited an average AUC, accuracy, sensitivity, and specificity of 0.81, 0.79, 0.61, and 0.86 upon internal validation. In contrast, the cut1 model achieved an average AUC, accuracy, sensitivity, and specificity of 0.85, 0.82, 0.72, and 0.86, respectively. Notably, as compared to the dome model, the cut1 model demonstrated significant enhancements in the first three evaluation metrics.

For external validation, we utilized an independent project within the AneuX morphology database, the @neurIST project, which comprises 87 ruptured and 72 unruptured aneurysms. It is noteworthy that there were noticeable dissimilarities in the composition of ruptured and unruptured aneurysms between the external and internal validation set. Application of the trained DL weights to the external validation set resulted in the dome model achieving an average AUC of 0.69, while the cut1 model attained an average AUC of 0.71. Consequently, the cut1 model outperforms the dome model in the external validation set as well.

According to the literature (Juchler et al., 2022), the authors of the original dataset developed a PCA-based LASSO regression model, which achieved an average AUC of 0.82 in internal validation and 0.67 in external validation. In comparison, our cut1 model exhibited superior performance both in terms of internal validation and generalizability.

Nonetheless, we observed that both the dome and cut1 models demonstrated relatively lower sensitivity compared to other metrics. For instance, in the cut1 model, the mean accuracy was 0.82, mean specificity was 0.86, while the mean sensitivity was 0.72. We have deliberated upon these issues, and possible explanations will be further elucidated in the discussion section.

## 4 Discussion

The utilization of DL methodologies in the realm of cerebrovascular disease imaging studies is expanding dramatically, including various aspects such as detection, prediction, and treatment. Nevertheless, the lack of sufficient clinical data has hindered the progress of DL in assessing the risk of IA rupture. This study aimed to assess the viability and effectiveness of a point cloud-based DL model for predicting the likelihood of IA rupture. To achieve this, we utilized an open-source, multi-centric database.

During the preprocessing of the cut1 model, we ensured the preservation of both the aneurysm dome and a portion of the parent vessels. This preservation facilitated the DL model to extract a wide range of morphological features associated with both ruptured and unruptured aneurysms. It also allowed for the analysis of structural aspects between the aneurysm dome and parent vessels, such as the aneurysm-to-vessel size ratio, which has been demonstrated to be a critical parameter in assessing aneurysm rupture risk (Dhar et al., 2008). Furthermore, the presence of small vessel branches on parent vessels may introduce redundant information into the model, increasing its complexity. Managing a large number of input features complicates the process of extracting key features, increases execution time, and has the potential to impede model convergence (Visalakshi and Radha, 2014). Therefore, we chose to remove the smaller branches attached to the parent vessels deliberately in order to reduce model complexity and enhance computational efficiency.

Given that a point cloud is essentially a set of dense 3D points, each possessing its own coordinates in 3D space. During the sampling process, it is critical to minimize the loss of essential information while maintaining sufficient morphological details of aneurysms. Hence, after comparing the need for a detailed representation against the computational cost, we chose a high-density sampling of 8,192 points to ensure that the precise spatial contour and morphological features of aneurysms could be conveyed. The sampled point cloud model is depicted visually in Figure 2B.

The performance of our approach was shown to be superior when compared to the PCA-based LASSO regression model. However, as the authors of the original dataset noted, we ran into problems with the model’s generalizability when conducting external validation using a novel and heterogeneous dataset. Additionally, we have observed a phenomenon in which both models exhibit favorable accuracy and specificity, while displaying relatively lower sensitivity.

Two potential issues with the dataset could be contributing to the model’s lack of generalizability. Firstly, the composition ratio of ruptured and unruptured aneurysms differed significantly across the HUG and the @neurIST projects. Specifically, ruptured aneurysms accounted for approximately 27% of all aneurysms in the HUG project, while they made up around 55% in the @neurIST project. This structural composition disparity between the external validation set and the training set can have an effect on the model’s generalizability. Furthermore, we found discrepancies in resolution and artifacts in the original 3D vessel models from the two projects. Despite efforts to minimize these discrepancies during preprocessing by utilizing “smooth” and “remesh” techniques, the potential variation in data quality between the external validation set and the training set could hamper the model’s generalizability in DL.

In this study, we proposed a possible explanation for the observed discrepancy between the model’s high levels of accuracy and specificity and its relatively low sensitivity. The term “sensitivity” refers to the model’s ability to reliably identify ruptured aneurysms among the total number of true ruptured aneurysms, as expressed by the ratio of true positive predictions (correctly identified ruptured aneurysms) to actual ruptured aneurysms in the current study. It is worth noting that both the HUG and @neurIST projects primarily included aneurysms imaged with 3DRA, which is commonly used in the context of clinical interventions (van Rooij et al., 2009). However, it is vital to recognize that this reliance on 3DRA creates a potential selection bias towards unruptured aneurysms within the database. Specifically, unruptured aneurysms in the database are more likely to meet the criteria for intervention and subsequently undergo clinical interventions following 3DRA. As a result, the database may contain a higher proportion of large-sized and irregularly-shaped unruptured aneurysms compared to what is typically encountered in real-world scenarios. This disparity in size and morphology between the database and the actual population of unruptured aneurysms could plausibly account for the lower sensitivity.

We evaluated the performance of the optimal curve using the external validation set, and the risk score visualization for three examples is depicted in Figure 4. The first aneurysm exhibited irregular morphology, with a high aspect ratio and size ratio, along with the presence of a noticeable bulge. The aneurysm actually ruptured, and our model predicted a risk score of 0.982. The second aneurysm had a regular shape and a smooth surface. The aneurysm did not rupture, and our model predicted a risk score of 0.089. Additionally, the third aneurysm, similar to the first one, presented irregular morphology, with a high aspect ratio and size ratio, accompanied by a noticeable bulge. Although the aneurysm did not rupture in reality, based on clinical experience, it was deemed to have a high risk of rupture, which aligns with the model’s predicted risk score of 0.933. These results demonstrate that the point cloud representation can effectively capture the contours and morphological features of aneurysms.

Our study has certain limitations. Firstly, IA rupture is a complex event influenced by multiple factors. Some clinical risk factors, such as blood pressure and smoking history, as well as hemodynamic factors such as wall shear stress and oscillatory shear index, which might potentially improve the model’s performance, were not included in the model due to data availability and technical obstacles. Secondly, while earlier studies (Kataoka et al., 2000; Beck et al., 2003; Lindgren et al., 2016) indicated that the morphology of IA did not change considerably before and after rupture, evaluating rupture risk based on rupture and non-rupture events may introduce some error into the experimental results. Furthermore, our study did not validate the performance differences between PointNet and PointNet++, even if the latter is an iterative version of former. Hence, we cannot definitively conclude that PointNet++ necessarily outperforms PointNet in our study. Finally, our study only used classical algorithms for point clouds. It is worth noting that while classical networks are widely recognized and accepted, recent research has introduced novel DL algorithms based on point clouds, such as physics-informed neural networks (PINNs) (Zhang et al., 2023) and point cloud transformer (PCT) (Guo et al., 2021). These emerging algorithms may perform better in certain tasks. Given these limitations, future studies should incorporate multiple variables associated with rupture risk in prospective, multicenter follow-up studies and validate the latest point clouds-based DL algorithms, with the goal of providing a more comprehensive assessment of the rupture risk for both stable and unstable aneurysms.

## 5 Conclusion

Our research evaluated the feasibility and efficacy of a point cloud-based DL model in predicting the likelihood of IA rupture using a prospective, multi-centric dataset, indicating that point cloud, as a 3D visualization tool for IA, can effectively capture the spatial contour and morphological aspects of aneurysms. In addition, we examined the performance of two models with distinct cropping procedures, highlighting the importance of structural elements between the dome and parent vessels. The point cloud-based DL model exhibited good performance in predicting aneurysm rupture risk while also facing challenges in generalizability.

## Data Availability

The data presented in the study are deposited in the zenodo repository, accession DOI: 10.5281/zenodo.6678442.

## References

[B1] An X. He J. Di Y. Wang M. Luo B. Huang Y. (2022). Intracranial aneurysm rupture risk estimation with multidimensional feature fusion. Front. Neurosci. 16, 813056. 10.3389/fnins.2022.813056 35250455 PMC8893318

[B2] Aneurisk-Team (2012). AneuriskWeb project website. United States: Emory University, Department of Math & CS.

[B3] Beck J. Rohde S. el Beltagy M. Zimmermann M. Berkefeld J. Seifert V. (2003). Difference in configuration of ruptured and unruptured intracranial aneurysms determined by biplanar digital subtraction angiography. Acta Neurochir. 145, 861–865. 10.1007/s00701-003-0124-0 14577007

[B4] Berti G. Hose R. Marzo A. Villa-Uriol M.-C. Singh P. Lawford P. (2010). Integrated biomedical informatics for the management of cerebral aneurysms - D23v2 - analysis protocols version 2. Aneurist. Available at: http://www.aneurist.org/UserFiles/File/PUBLIC_DELIVERABLES/D23v2_v1.2_final.pdf (Accessed April 12, 2022).

[B5] Bizjak Ž. Pernuš F. Špiclin Ž. (2021). Deep shape features for predicting future intracranial aneurysm growth. Front. Physiol. 12, 644349. 10.3389/fphys.2021.644349 34276391 PMC8281925

[B6] Brown R. D. Broderick J. P. (2014). Unruptured intracranial aneurysms: epidemiology, natural history, management options, and familial screening. Lancet Neurol. 13, 393–404. 10.1016/S1474-4422(14)70015-8 24646873

[B7] Can A. Castro V. M. Ozdemir Y. H. Dagen S. Yu S. Dligach D. (2017). Association of intracranial aneurysm rupture with smoking duration, intensity, and cessation. Neurology 89, 1408–1415. 10.1212/WNL.0000000000004419 28855408 PMC5649762

[B8] Chen R. Mo X. Chen Z. Feng P. Li H. (2022b). An integrated model combining machine learning and deep learning algorithms for classification of rupture status of IAs. Front. Neurol. 13, 868395. 10.3389/fneur.2022.868395 35645962 PMC9133352

[B9] Chen X. Lei Y. Su J. Yang H. Ni W. Yu J. (2022a). A review of artificial intelligence in cerebrovascular disease imaging: applications and challenges. Curr. Neuropharmacol. 20, 1359–1382. 10.2174/1570159X19666211108141446 34749621 PMC9881077

[B10] de Boer P.-T. Kroese D. P. Mannor S. Rubinstein R. Y. (2005). A tutorial on the cross-entropy method. Ann. operations Res. 134, 19–67. 10.1007/s10479-005-5724-z

[B11] Dhar S. Tremmel M. Mocco J. Kim M. Yamamoto J. Siddiqui A. H. (2008). Morphology parameters for intracranial aneurysm rupture risk assessment. Neurosurgery 63, 185–196. 10.1227/01.NEU.0000316847.64140.81 18797347 PMC2570753

[B12] Goodfellow L. Bengio Y. Courville A. (2016). Deep learning. United States: MIT Press, 180–184.

[B13] Greving J. P. Wermer M. J. H. Brown R. D. Morita A. Juvela S. Yonekura M. (2014). Development of the PHASES score for prediction of risk of rupture of intracranial aneurysms: a pooled analysis of six prospective cohort studies. Lancet Neurol. 13, 59–66. 10.1016/S1474-4422(13)70263-1 24290159

[B14] Guo M. Cai J. Liu Z. Mu T. Martin R. Hu S. (2021b). PCT: point cloud transformer. Comp. Vis. Media 7, 187–199. 10.1007/s41095-021-0229-5

[B15] Guo Y. Wang H. Hu Q. Liu H. Liu L. Bennamoun M. (2021a). Deep learning for 3D point clouds: a survey. IEEE Trans. Pattern Anal. Mach. Intell. 43, 4338–4364. 10.1109/TPAMI.2020.3005434 32750799

[B16] Harris C. R. Millman K. J. van der Walt S. J. Gommers R. Virtanen P. Cournapeau D. (2020). Array programming with NumPy. Nature 585, 357–362. 10.1038/s41586-020-2649-2 32939066 PMC7759461

[B17] Juchler N. Schilling S. Bijlenga P. Kurtcuoglu V. Hirsch S. (2022). Shape trumps size: image-based morphological analysis reveals that the 3D shape discriminates intracranial aneurysm disease status better than aneurysm size. Front. Neurol. 13, 809391. 10.3389/fneur.2022.809391 35592468 PMC9110927

[B18] Kashiwazaki D. Kuroda S. Sapporo SAH Study Group (2013). Size ratio can highly predict rupture risk in intracranial small (<5 mm) aneurysms. Stroke 44, 2169–2173. 10.1161/STROKEAHA.113.001138 23743979

[B19] Kataoka K. Taneda M. Asai T. Yamada Y. (2000). Difference in nature of ruptured and unruptured cerebral aneurysms. Lancet 355, 203. 10.1016/S0140-6736(99)03881-7 10675125

[B20] Kim H. C. Rhim J. K. Ahn J. H. Park J. J. Moon J. U. Hong E. P. (2019). Machine learning application for rupture risk assessment in small-sized intracranial aneurysm. J. Clin. Med. 8, E683. 10.3390/jcm8050683 PMC6572384 31096607

[B21] Kleinloog R. de Mul N. Verweij B. H. Post J. A. Rinkel G. J. E. Ruigrok Y. M. (2018). Risk factors for intracranial aneurysm rupture: a systematic review. Neurosurgery 82, 431–440. 10.1093/neuros/nyx238 28498930

[B22] Li G. Song X. Wang H. Liu S. Ji J. Guo Y. (2021b). Prediction of cerebral aneurysm hemodynamics with porous-medium models of flow-diverting Stents via deep learning. Front. Physiol. 12, 733444. 10.3389/fphys.2021.733444 34603085 PMC8484706

[B23] Li G. Wang H. Zhang M. Tupin S. Qiao A. Liu Y. (2021a). Prediction of 3D Cardiovascular hemodynamics before and after coronary artery bypass surgery via deep learning. Commun. Biol. 4, 99. 10.1038/s42003-020-01638-1 33483602 PMC7822810

[B24] Lindgren A. E. Koivisto T. Björkman J. von Und Zu Fraunberg M. Helin K. Jääskeläinen J. E. (2016). Irregular shape of intracranial aneurysm indicates rupture risk irrespective of size in a population-based cohort. Stroke 47, 1219–1226. 10.1161/STROKEAHA.115.012404 27073241

[B25] Morita A. Kirino T. Hashi K. Aoki N. Fukuhara S. Hashimoto N. (2012). The natural course of unruptured cerebral aneurysms in a Japanese cohort. N. Engl. J. Med. 366, 2474–2482. 10.1056/NEJMoa1113260 22738097

[B26] Ou C. Li C. Qian Y. Duan C. Si W. Zhang X. (2022). Morphology-aware multi-source fusion-based intracranial aneurysms rupture prediction. Eur. Radiol. 32, 5633–5641. 10.1007/s00330-022-08608-7 35182202

[B27] Pedregosa F. Varoquaux G. Gramfort A. Michel V. Thirion B. Grisel O. (2011). Scikit-learn Mach. Learn. Python 12, 2825–2830. 10.48550/arXiv.1201.0490

[B28] Qi C. R. Su H. Kaichun M. Guibas L. J. (2017a). “PointNet: deep learning on point sets for 3D classification and segmentation,” in 2017 IEEE Conference on Computer Vision and Pattern Recognition, Honolulu, HI, USA, July 21 2017 to July 26 2017, 77–85.

[B29] Qi C. R. Yi L. Su H. Guibas L. J. (2017b). “PointNet++: deep hierarchical feature learning on point sets in a metric space,” in In Advances in neural information processing systems (United States: MIT Press), 5099–5108. 10.48550/arXiv.1706.02413

[B30] Reimers N. Gurevych I. (2017). “Reporting score distributions makes a difference: performance study of LSTM-networks for sequence tagging,” in Proceedings of the 2017 Conference on Empirical Methods in Natural Language Processing, Øksnehallen, Copenhagen, Denmark, September 7-11, 2017, 338–348.

[B31] Tada Y. Wada K. Shimada K. Makino H. Liang E. I. Murakami S. (2014). Roles of hypertension in the rupture of intracranial aneurysms. Stroke 45, 579–586. 10.1161/STROKEAHA.113.003072 24370755 PMC3935821

[B32] Takao H. Murayama Y. Otsuka S. Qian Y. Mohamed A. Masuda S. (2012). Hemodynamic differences between unruptured and ruptured intracranial aneurysms during observation. Stroke 43, 1436–1439. 10.1161/STROKEAHA.111.640995 22363053

[B33] Ujiie H. Tamano Y. Sasaki K. Hori T. (2001). Is the aspect ratio a reliable index for predicting the rupture of a saccular aneurysm? Neurosurgery 48, 495–502. 10.1097/00006123-200103000-00007 11270538

[B34] van Rooij S. B. T. van Rooij W. J. Sluzewski M. Sprengers M. E. S. (2009). Fenestrations of intracranial arteries detected with 3D rotational angiography. AJNR Am. J. Neuroradiol. 30, 1347–1350. 10.3174/ajnr.A1563 19439481 PMC7051541

[B35] Villa-Uriol M. C. Berti G. Hose D. R. Marzo A. Chiarini A. Penrose J. (2011). @neurIST complex information processing toolchain for the integrated management of cerebral aneurysms. Interface Focus 1, 308–319. 10.1098/rsfs.2010.0033 22670202 PMC3262441

[B36] Visalakshi S. Radha V. (2014). “A literature review of feature selection techniques and applications: review of feature selection in data mining,” in 2014 IEEE International Conference on Computational Intelligence and Computing Research, Coimbatore, India, 18-20 December 2014, 1–6.

[B37] Vlak M. H. M. Algra A. Brandenburg R. Rinkel G. J. E. (2011). Prevalence of unruptured intracranial aneurysms, with emphasis on sex, age, comorbidity, country, and time period: a systematic review and meta-analysis. Lancet Neurol. 10, 626–636. 10.1016/S1474-4422(11)70109-0 21641282

[B38] Wiebers D. O. Whisnant J. P. Huston J. Meissner I. Brown R. D. Piepgras D. G. (2003). Unruptured intracranial aneurysms: natural history, clinical outcome, and risks of surgical and endovascular treatment. Lancet 362, 103–110. 10.1016/s0140-6736(03)13860-3 12867109

[B39] Xiang J. Natarajan S. K. Tremmel M. Ma D. Mocco J. Hopkins L. N. (2011). Hemodynamic-morphologic discriminants for intracranial aneurysm rupture. Stroke 42, 144–152. 10.1161/STROKEAHA.110.592923 21106956 PMC3021316

[B40] Yang H. Cho K. C. Kim J. J. Kim J. H. Kim Y. B. Oh J. H. (2023). Rupture risk prediction of cerebral aneurysms using a novel convolutional neural network-based deep learning model. J. Neurointerv Surg. 15, 200–204. 10.1136/neurintsurg-2021-018551 35140167

[B41] Zhang X. Mao B. Che Y. Kang J. Luo M. Qiao A. (2023). Physics-informed neural networks (PINNs) for 4D hemodynamics prediction: an investigation of optimal framework based on vascular morphology. Comput. Biol. Med. 164, 107287. 10.1016/j.compbiomed.2023.107287 37536096

