# Supplementary material for: Assessment of intracranial aneurysm rupture risk using a point cloud-based deep learning model
Source: Front Physiol. 2024 Feb 15;15:1293380. doi: 10.3389/fphys.2024.1293380 (PMC10901972; doi:10.3389/fphys.2024.1293380)
Supplement: Supplementary file 1 [file Table1.DOCX]

Supplementary Material

**Assessment of Intracranial Aneurysm Rupture Risk Using a Point Cloud-based Deep Learning Model**

Heshan Cao*, Hui Zeng*, Lei Lv, Qi Wang, Hua Ouyang, Long Gui, Ping Hua, Songran Yang

*** Correspondence:**

Ping Hua: [huaping@mail.sysu.edu.cn](mailto:huaping@mail.sysu.edu.cn)

Songran Yang: [yangsr@mail.sysu.edu.cn](mailto:yangsr@mail.sysu.edu.cn)

**Supplementary** **Table 1** Five groups (eleven) repeated aneurysm models discovered during the preprocessing phase.

| Group | Name |
| --- | --- |
| 1 | SNF00000189_1sib |
|  | USFD_UNIGE_0037 |
| 2 | SNF00000556 |
|  | USFD_UNIGE_0033 |
| 3 | SNF00000055 |
|  | USFD_UNIGE_0001 |
| 4 | SNF00000449 |
|  | UPF_P0272.00_ID1 |
| 5 | SNF00000303 |
|  | ANSYS_UNIGE_33_631 |
|  | p527_Eg4ADxEGFh8cFwgOHAUMCgcW |

Note that the p series and SNF series belong to the HUG project, while the ANSYS series, UPF series, and USFD series belong to the @neurIST project.

**Supplementary** **Table 2** Unavailable rupture status for 15 aneurysm models.

| Number | Name |
| --- | --- |
| 1 | SNF00000071 |
| 2 | SNF00000127 |
| 3 | SNF00000134 |
| 4 | SNF00000137_01_1 |
| 5 | SNF00000137_01_2 |
| 6 | SNF00000155 |
| 7 | SNF00000227 |
| 8 | SNF00000244 |
| 9 | SNF00000316 |
| 10 | SNF00000360_01_1 |
| 11 | SNF00000360_01_2 |
| 12 | SNF00000419 |
| 13 | SNF00000439 |
| 14 | SNF00000547 |
| 15 | SNF00000605 |


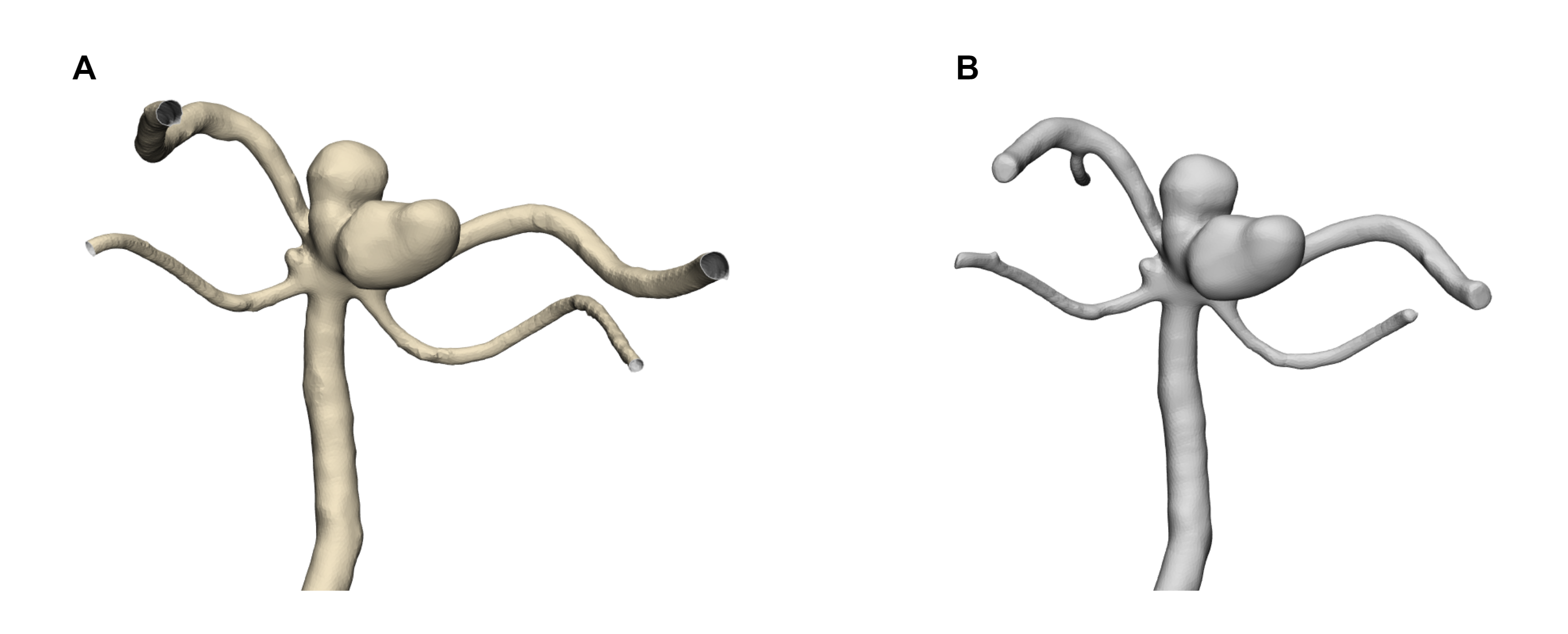


**Supplementary Figure 1.** A schematic diagram of one of the five-group repeated aneurysm models. **(A)** SNF00000189_1sib. (**B**) USFD_UNIGE_0037.
